# Individual Variation in Philopatry Is Unrelated to Activity and Space Use in the Undulate Skate *Raja undulata*

**DOI:** 10.1002/ece3.72135

**Published:** 2025-12-10

**Authors:** Alina Hillinger, David Villegas‐Ríos, Christopher T. Monk, Kenn Papadopoulo, Gonzalo Mucientes, Alexandre Alonso‐Fernández

**Affiliations:** ^1^ Doctoral Programme “Marine Science, Technology and Management” (DOMAR) University of Vigo Vigo Spain; ^2^ Instituto de Investigaciones Marinas (IIM‐CSIC) Vigo Spain; ^3^ GEOMAR Helmholtz Centre for Ocean Research Kiel Kiel Germany; ^4^ Centro de Investigação em Biodiversidade e Recursos Genéticos, CIBIO‐InBIO Universidade do Porto Vairão Portugal

**Keywords:** acoustic telemetry, animal behavior, behavioral syndrome, elasmobranch, *Raja undulata*

## Abstract

Individual variation in behavior is widespread in animals. Movement traits, which describe how individuals interact with their environment, can vary across a range of spatial and temporal scales. Much research has focused on understanding individual variation in movements at large spatio‐temporal scales, such as migration and dispersal, which are driven by, for example, size, age, feeding strategy, and reproductive needs. Recently, bio‐logging revealed individual variation in traits at finer scales that respond to day‐to‐day motives (e.g., foraging, predator avoidance) such as activity, chronotype, or space use. As traits operating at different scales may respond to different mechanisms, an important yet unresolved question is whether behavioral traits covary across spatial and temporal scales. Answering this may shed light on fundamental aspects of behavioral variation and regulation in animals. We addressed this gap by acoustically tracking 179 individual *Raja undulata* over four years in a coastal archipelago in NW Spain. Using daily presence‐absence data, we classified individuals into three philopatry patterns: continuous residency, seasonal residency, or site fidelity. We then used a multivariate mixed modeling approach to explore how activity space size (km^2^) and activity rate (m/h) varied among individuals and their relationship with philopatry patterns. We found that activity and space use covaried and were consistently different among individuals but were unrelated to philopatry, suggesting that they represent independent axes of behavior. We highlighted that behavioral variation might carry eco‐evolutionary implications through selection, with selective pressures acting on philopatry being unlikely to drive co‐evolution of activity or space use.

## Graphical abstract

Consistent individual variation in fine‐scale movement traits is unrelated to movements occurring at larger spatio‐temporal scales in undulate skates ( *Raja undulata* ).

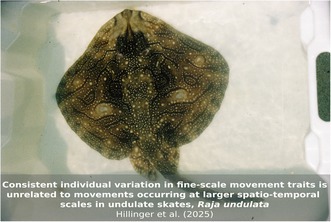

## 1 Introduction

Large‐scale ecological processes are the collective outcome of many individual movements and decisions made by animals (Chapman et al. 2012; Sih et al. 2004). Animal movement links individual behavior and population dynamics and connects individuals with their environment (Campos‐Candela et al. 2019; Nathan et al. 2008; Spiegel et al. 2017). Consequently, gaining a comprehensive understanding of animal movement and drivers thereof is central to informing conservation and management (Espinoza et al. 2021; Spiegel et al. 2017). For example, knowledge on the habitat use of a species allows one to assess whether spatial management measures match population needs (Graham et al. 2012), while information about corridors, movement routes, or migrations can identify the spatio‐temporal scales at which conservation and management measures are most beneficial (Brownscombe et al. 2019; Croft‐White et al. 2023).

In nature, animals can move across vast spatial and temporal scales. A vast body of research has previously assessed individual differences in movement traits at large spatial and temporal scales, such as dispersal (Cote et al. 2010; Nanninga and Berumen 2014), philopatry (Campbell et al. 2012; Flowers et al. 2016), migration strategy (Chapman et al. 2012) and timing (Abrahms et al. 2018; Jensen et al. 2020). However, in recent decades, biologging methods have enabled an unprecedented understanding of animal behaviors at finer scales (e.g., daily or weekly), revealing, in many cases, substantial variation among individuals (Krause et al. 2013; Nathan et al. 2008). Consistent among‐individual variation in behavioral traits across contexts and time (termed “animal personality”) is prominent within animal populations (Bell et al. 2009; Maggs et al. 2019), and movement traits are not an exception (Stuber et al. 2022). For example, individual variation explained 30 to 83% of differences seen in awakening time, rest onset, log‐rest duration, and traveled distance in Pearly razorfish ( *Xyrichtys novacula* ) (Alós et al. 2017). Likewise, individual identity in Atlantic cod ( *Gadus morhua* ) accounted for 17%, 66%, and 67% of variation in diel vertical migration, activity, and activity space size, respectively (Monk et al. 2023).

Movement traits may be correlated across different spatio‐temporal scales, as all movements are based on behavioral decisions made by individual animals (Chapman et al. 2012; Sih et al. 2004; Stuber et al. 2022). These decisions are driven by a range of internal (e.g., fitness, sex, personality, capacity to navigate, capacity to move) and external factors (e.g., social factors, abiotic factors, environment, habitat) (Michelangeli et al. 2022; Nathan et al. 2008; Spiegel et al. 2017). Correlated behavioral traits, referred to as behavioral syndromes, have been observed in many species and traits (Conrad et al. 2011; Sih et al. 2004, 2012), including movement traits (Harrison et al. 2015; Spiegel et al. 2017). In the marine realm, the question of whether movement behaviors that occur at different spatial and temporal scales are related to each other remains largely unresolved. We hypothesize that large‐scale movement behaviors, such as philopatry or partial migration, are linked to small‐scale behaviors like activity, space use, and foraging. We largely base this hypothesis on the *Pace‐of‐life syndrome (POLS) hypothesis* (Réale 2010), which links fast‐paced, bold individuals to larger‐scale movements, as these individuals tend to be more exploratory and have larger home ranges. Our hypothesis is further supported by previous literature, such as Chapman, Hulthén, et al. (2011), who found that in fish, bold individuals are more likely to migrate, suggesting that traits like boldness may be correlated with larger‐scale movement patterns. Furthermore, the behavioral trait boldness has previously been found to be positively correlated with activity in fish (Axling et al. 2023; Malorey et al. 2024), suggesting potential links between our chosen small‐scale behaviors and large‐scale behaviors including migration or philopatry. Similarly, exploration was found to be more pronounced in migratory species compared to resident ones (Mettke‐Hofmann et al. 2005), reinforcing the idea that smaller‐scale behaviors may influence larger‐scale migratory patterns. Energetic status may also play a role, as fish with higher energy reserves have a higher tendency to migrate and be more active (Shry et al. 2019). Together, these findings highlight that small‐scale behaviors, including activity and space use, may be key factors in shaping larger‐scale movement decisions like migration and philopatry.

In this study, we examined potential links between philopatry, expressed at an inter‐annual scale, and two behaviors (activity and space use) measured at a much finer spatial and temporal scale, using the undulate skate *Raja undulata* (Lacepède, 1802) as a case study. The species is well suited as a model species, as individuals aggregate locally and spend long periods of time in a small area, enabling high‐resolution tracking. Importantly, the species previously showed large variation in residency patterns (Leeb et al. 2021), with individuals varying from highly resident to transient. In particular, we assessed (i) consistent among‐individual variation (i.e., repeatability) in fine‐scale movement traits as well as variation in philopatry patterns and (ii) links between behavioral traits expressed at different spatio‐temporal scales at the among‐individual level. We discuss the implications of skate behavior and links among behavioral traits for conservation, management, and evolution.

## 2 Materials and Methods

### 2.1 Study System

**FIGURE 1:**
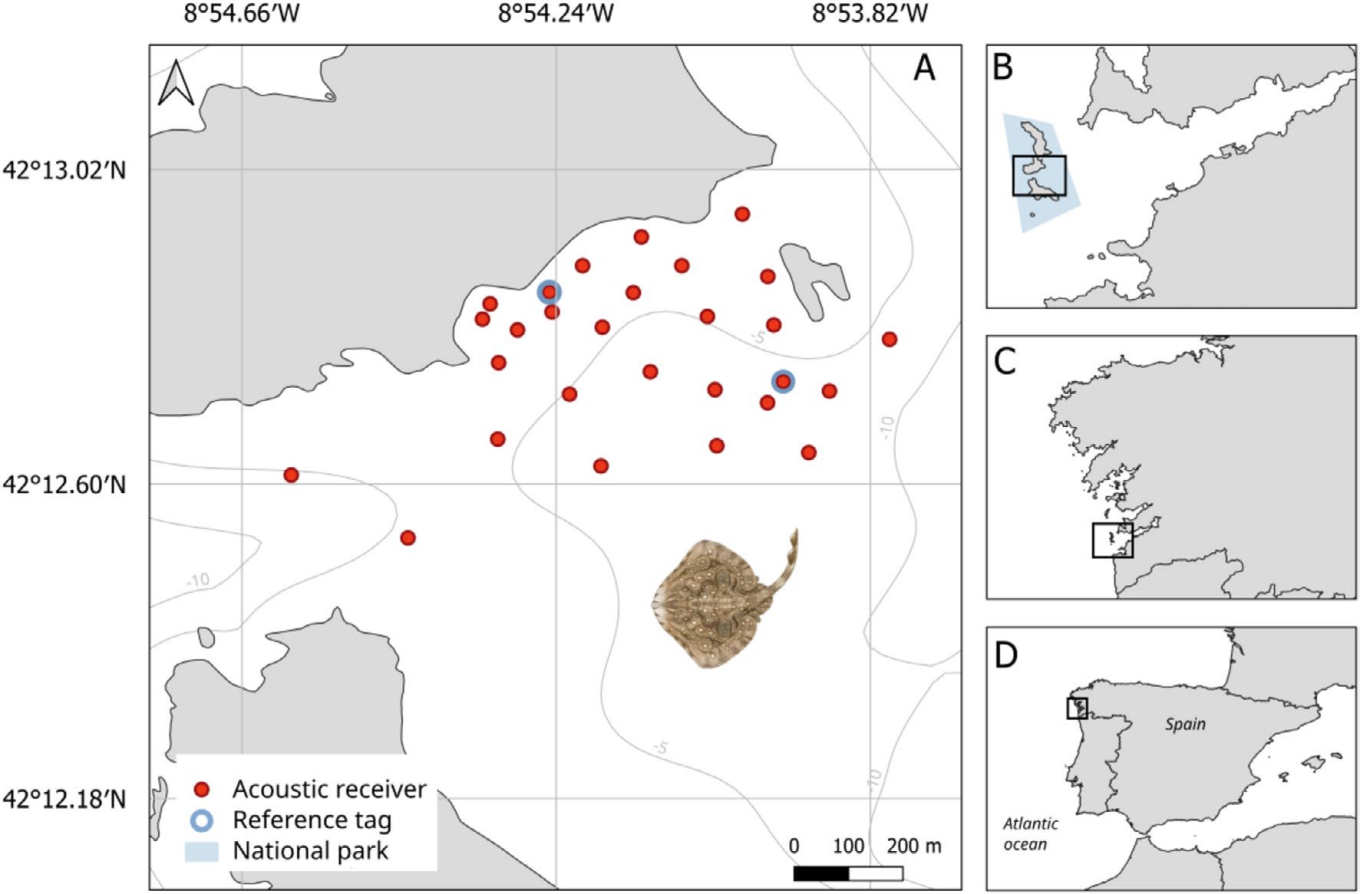
Detailed view of the acoustic telemetry array (A), the location of the Cíes Archipelago within the ‘Parque Nacional Marítimo Terrestre das Illas Atlánticas de Galicia’ (B), its location within Galicia (C), its location within Spain (D).

This research was carried out at the Cíes archipelago in Galicia (NW Spain) (Figure 1), which is part of the National Park ‘Parque Nacional Marítimo Terrestre das Illas Atlánticas de Galicia’. Here, recreational fishing is prohibited while commercial small‐scale fisheries are subject to gear restrictions (Xunta de Galicia 2019). The area where the study site is located is highly productive, due to upwelling events that typically occur in spring and summer (Álvarez‐Salgado et al. 2002). As a result, the study site and surrounding areas serve as important fishing grounds for the local artisanal fishing fleet, which supports the economy of one of the most fisheries reliant areas in Europe (Cambiè et al. 2012).

To investigate the behavior of *R. undulata* , we used an acoustic telemetry array of 28 receiver stations in the archipelago (Figure 1), covering an area of ~0.9 km^2^. Twenty‐four stations consisted of both Thelma Biotel (model TBR 700L) and Innovasea (previously VEMCO; model VR2W) acoustic receivers (due to a change of equipment manufacturers throughout the study period), and four stations of Thelma Biotel (model TBR 700L) acoustic receivers only. The seafloor inside the acoustic array is composed mainly of sand and rocky reefs. Consequently, all receivers were installed using auger anchors in sandy bottoms, except for two stations on hard substrates that used a weight from which they were drawn up with a buoy (Leeb et al. 2021). Based on previous range tests (see Leeb et al. 2021), receiver stations were placed at ~150 m distance, which allowed for overlap between detection ranges (Leeb et al. 2021; Mucientes et al. 2021). We deployed reference transmitters (*n* = 2 Innovasea [*n* = 1 V16‐4x‐069k‐2; *n* = 1 V13‐1x‐069k‐3] and *n* = 4 Thelma Biotel [*n* = 2 D‐2LP13; *n* = 2 D‐2LP9L]) at known positions in the array to estimate fish positioning error (AMIRIX Systems 2013; Payne et al. 2010). Synchronization transmitters were used to synchronize the internal clocks of receivers. Data download took place every six months. Receivers were installed at depths between 3.3 and 13.1 m.

### 2.2 Acoustic Tagging

We tagged 179 individual *R. undulata* throughout four years (*n* = 44 in 2019, *n* = 41 in 2020, *n* = 49 in 2021, *n* = 45 in 2022; Annex 1). All but two individuals were caught by hand while scuba diving inside the acoustic array. Skates were slowly (~3 m/min) brought to the surface inside a mesh net (Leeb et al. 2021). Two individuals were caught using bottom long‐lines by artisanal fishers. Individuals were anesthetized using 100 mg/L tricaine methane sulfonate (MS‐222) for ~2 to 3 min. Disc length and sex were recorded. Tagging was primarily internal (internal *n* = 167, external *n* = 8, internal and external *n* = 4). Transmitters were inserted through a small incision into the peritoneal cavity, which was then surgically closed using dissolving thread. Transmitters were attached externally with tags to the pectoral fin (anchored with monofilament). Transmitter size (9–16 mm diameter) varied according to body size, and transmitters had randomized signal transmission delays ranging from 40 to 160 s. Transmitters were either manufactured by Innovasea or Thelma Biotel. To recognize acoustically tagged individuals in case of recaptures, skates were also marked with t‐bar plastic tags (Floy Tag). Individuals recovered in saltwater tanks before being released onto the seafloor at the site of capture. To explore large‐scale movements along the coast through recaptures, 40 additional individuals were marked with t‐bar tags only.

### 2.3 Data Processing and Filtering

Acoustic data recording took place between May 5th 2019 and June 4th 2023, and recaptures were recorded until August 2024. Analyses of acoustic telemetry data were based on two data formats: detections (presence/absence) with linked depth records and estimated positions. Detection files were filtered to remove single detections of the same transmitter in a 24 h timespan (Meyer et al. 2007). In case of mortalities, determined by examining positions (based on centres of activity, COAs) and depth data (Villegas‐Ríos et al. 2020), detections post mortality were removed.

Triangulation‐based high spatial resolution Pinpoint (Thelma Biotel) and VPS (Innovasea) positions (hereafter collectively ‘positions’) were estimated by the manufacturers (Smith 2013; Thelma Biotel 2018). We stripped the dataset of positions with large spatial errors by visually assessing the trade‐off between estimated error (based on metrics of spatial error: horizontal Position Error (HPE, HPEm) for VPS data (Orrell et al. 2023; Smith 2013) and horizontal dilution of precision (HDOP) for Pinpoint data) and the number of retained positions (Freitas et al. 2016). We chose a cut‐off median error tolerance of < 3.5 m. The resulting dataset consisted of 83.83% (*n* = 951,402) of VPS and 88.25% (*n* = 95,912) of Pinpoint positions.

### 2.4 Estimation of Movement Traits

We estimated three movement traits for each individual: philopatry pattern, activity space size, and activity.

#### 2.4.1 Philopatry Pattern

**FIGURE 2:**
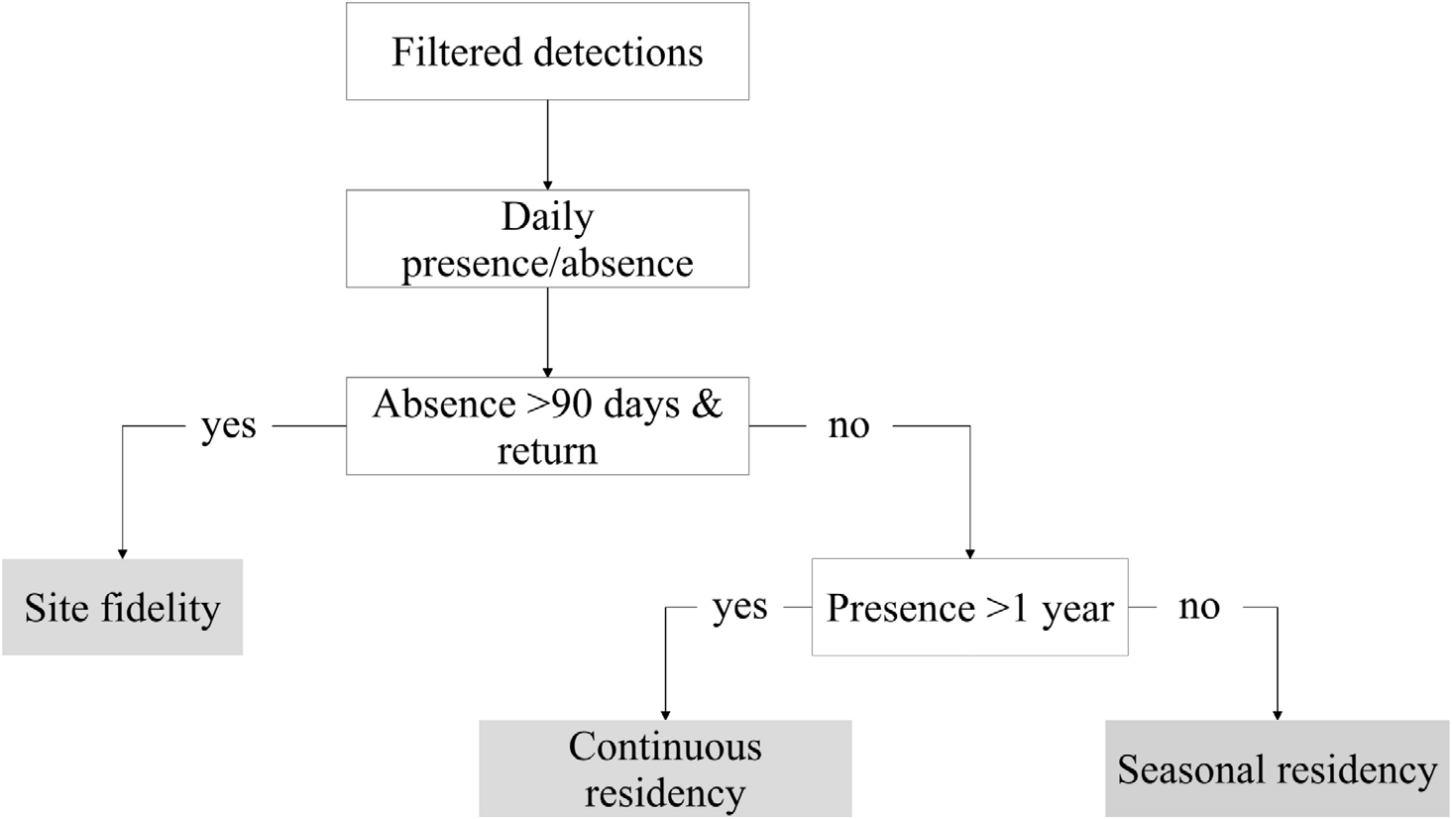
Philopatry pattern classification for individual *Raja undulata* based on daily presence/absence data.

In the context of this study, philopatry was defined as “individuals frequently returning to or staying in their home ranges, birthplaces, or other specific localities” (Flowers et al. 2016). Preliminary inspection of time series of daily presence and absence data suggested great variability in how individuals used the study area over time, with a tendency of *R. undulata* to aggregate in the study area during summer and disperse in winter (Annex 2 Figure S1), as observed in Leeb et al. (2021). Still, some individuals were observed to reside in the area year‐round. Following Chapman et al. (2015) and Flowers et al. (2016) philopatry patterns were classified as (Figure 2): (1) site fidelity, when an individual left the area for a period longer than 90 days and returned afterwards, (2) seasonal residency, when an individual stayed for less than a year displaying no absences longer than 90 days, and (3) continuous residency, when an individual stayed more than one year without absences longer than 90 days. Note that both continuous and seasonal residents are characterized by an uninterrupted (i.e., no absences longer than 90 days) stay in the study area followed by a dispersal with no return. We selected 90 days as the threshold to separate long‐term from short‐term absences (and hence site fidelity from residency) based on preliminary analysis. This included visual inspection of seasonal presence‐absence patterns to best reflect a seasonal trend of arrival and emigration as emphasized in return and exit dates of individuals (see Figure S1). A threshold of 90 days was further selected to account for individuals potentially being just outside of the acoustic telemetry array, thus not representing “true” exits from the area, and discard all exits that may be part of the daily short‐termed excursions to areas just outside of the array.

To assess the impact of the 90‐day threshold used to assign philopatry patterns, we conducted a sensitivity analysis by fitting the model on a random subset of the complete data set (*n* = 700 rows) using alternative absence thresholds of 60 and 120 days. The results showed minimal differences in both the number of animals classified under each philopatry pattern and in the model outputs (Annex 2 Table S1, Figure S2). Therefore, we retained the 90‐day threshold for the final model, as it did not substantially affect the classification or the model results.

#### 2.4.2 Activity Space Size

Individual activity space size (km^2^) during day and night was estimated as the weekly 95% kernel utilization distribution (KUD, split into day‐time and night‐time) based on positions using the *adehabitatHR* package (version 0.4.21) using a smoothing factor of 50 in R (Calenge 2020). Sunrise and sunset times were used to separate day‐time and night‐time using the *suncalc* package (version 0.5.1) (Thieurmel and Elmarhraoui 2022). To reduce potential bias, KUDs were only estimated if > 4 position‐days were available per individual per week (successive and non‐successive days) (Leeb et al. 2021).

#### 2.4.3 Activity

Individual trajectories were produced from time‐series of consecutive positions using the *adehabitatLT* library (version 0.3.25) in R (Calenge 2020). To distinguish fish being undetected inside the study area (e.g., resting under sand) from absent fish, individual trajectories were split into sub‐trajectories using a selective flow chart (Annex 2 Figure S3) as adapted from Leeb et al. (2021). Time series of positions were then interpolated every 80 s (i.e., the minimum transmission delay for all but 10 transmitters). We estimated activity (m/h) as follows:

(1) $$\text{activity}=\frac{\text{dist}}{\mathrm{dt}}\times 3600$$

with distance (*dist*) being the distance between two successive positions and *dt* the interpolation period of 80 s. The activity dataset was then aggregated by week, separately for day‐time and night‐time, to perform analyses at the same temporal scale as activity space size.

### 2.5 Data Analysis

To investigate consistent individual‐level variation in fine‐scale movement behaviors and covariation between them, we used a Bayesian multivariate linear mixed‐effects model. Models were fitted in R, using the *MCMCglmm* package (version 2.36) and Markov chain Monte Carlo (MCMC) techniques (Hadfield 2010). As previous knowledge about relationships was unavailable, all models were fitted using an uninformative inverse‐Wishart prior (R: V = diag(2), nu = 1.002, G: V = diag(2), nu = 1.002) for the residual and random variance components, respectively, and default priors for fixed effects, assuming a “gaussian” family (Hadfield 2010). The model included two response variables: activity space size and activity, as continuous variables measured repeatedly for each individual. Both variables were log‐transformed to meet normality assumptions. To assess whether there was an effect of the philopatry pattern on activity and/or activity space size, philopatry was added as a predictor variable. Disc length (DL, in cm), sex (male vs. female), week (numerical from 1 to 53) and daytime (day vs. night) were included as fixed effects. The identity of each skate was included as a random effect. We included predictors based on their anticipated ecological relevance. We assumed linear relationships for the fixed effects except for week. Week was fitted as third‐order polynomial to account for any non‐linear effect due to potential seasonal environmental effects on activity space size and activity. Models were run with four iteration chains for 1,000,000 iterations, with a burn‐in of 50,000 and thinning length of 200. We applied the “autocorr” function from the *coda* package (version 0.19‐4.1, Plummer et al. 2024) and the “afc” function (*stats* package version 4.3.3, R Core Team 2024) to assess autocorrelation in the Markov chains. Model convergence was assessed visually using trace plots (Annex 2 Figure S4) and using the Gelman‐Rubin statistic (values were < 1.1, indicating good convergence, Du et al. 2022). The effects of different predictors on the response variables were assessed based on 95% confidence intervals of the posterior distributions and deemed significant if confidence intervals did not include zero. The correlation among the two fine‐scale movement traits, activity and activity space, was estimated from the median and 95% credible intervals of the posterior distribution of among‐individual covariance, normalized by the product of the square root of the among‐individual variances of both traits (Houslay and Wilson 2017).

Adjusted repeatability (*r*_adj_) was then computed to investigate consistency among‐individual variation at the individual level of activity space size and activity following Equation (2):

(2) $$r_{\mathrm{adj}}=\frac{V_{\mathrm{ind}}}{V_{\mathrm{ind}}+V_{\mathrm{res}}}$$

where *V*_ind_ describes the among‐individual variance and *V*_res_ describes the within‐individual variance, thus the total variance would be *V*_total_ = *V*_ind_ *+ V*_res_. We then assessed the 95% credible interval of the posterior of the adjusted repeatability. As we were not able to account for temporal autocorrelation in our model (a restriction of *MCMCglmm*), we followed Villegas‐Ríos et al. (2017) and based our repeatability estimates on weekly measures, which, compared to monthly measures, yield a lesser effect of temporal autocorrelation.

## 3 Results

Over four years, a total of 1,137,583 acoustic detections (after filtration) were recorded from 179 tagged *R. undulata* . Of the tagged individuals, 73 were females (40.1%) and 106 were males (59.9%). Individuals ranged between 24 and 59 cm in disc length (females: 26–59 cm, mean = 41.27 cm; males: 24–57 cm, mean = 40.87 cm). There were a total of 29 recaptures of telemetry‐tagged individuals during the study period, and another 6 recaptures of individuals that were tagged with only t‐bars. The linear distance between mark and recapture locations ranged from a few meters (i.e., inside the study area) to 52 km (female, 44 cm DL; Annex 2 Figure S5, Annex 1). The maximum interval between mark and recapture was 1067 days (01.06.2021–02.05.2024; spanning four tagging years). One individual was retrieved dead from an abandoned net on the northwestern boundary of the study site 103 days after tagging. A total of 12 individuals were classified as having experienced mortality, five based on reported fisheries recaptures and the remaining seven based on depth and location data from telemetry tags. One of these mortalities occurred within 24 h after tagging, and the individual was removed from the analysis, as data may not represent natural behavior. One externally attached transmitter yielded no data due to malfunction, and the individual was removed from the analysis. Therefore, our final dataset included 177 individuals.

### 3.1 Philopatry Patterns

**FIGURE 3:**
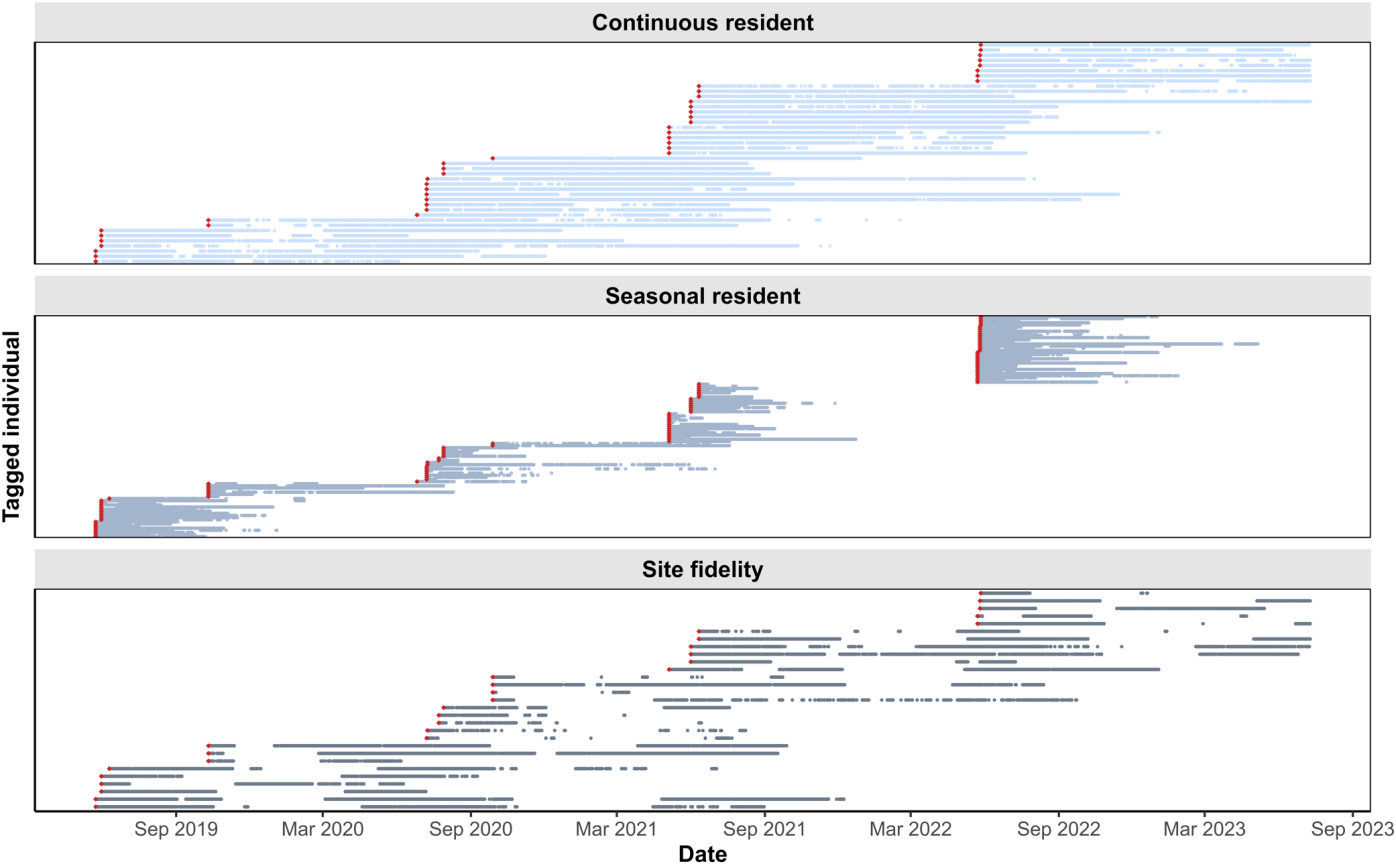
Presence‐absence plots of *Raja undulata* in the study area for three philopatry patterns: Continuous residency, seasonal residency and site fidelity. Red dots represent tagging dates, each horizontal line represents detections of one acoustically tagged individual.

We found large variability in how individual *R. undulata* used the study area. Individuals were observed to be present between 4 and 906 days in the study area. Out of 177 individuals, 105 (59.3%) were classified as seasonal residents, 29 individuals (16.4%) showed site fidelity, and 43 individuals (24.3%) were found to be continuous residents (Figure 3). A seasonal trend in the timing of emigration was observed: individuals displaying site fidelity were absent mainly between November/December and March/April, while individuals displaying seasonal residency permanently emigrated between September and January (Figure 3). This supports our field observations and suggests a seasonal aggregation of *R. undulata* at our study site. No individual was detected in the array for more than three consecutive years; however, recaptures indicate that skates may visit the study area for at least four years (individual 1: male, 31 cm DL; individual 2: female, 40 cm DL; Annex 1).

### 3.2 Individual Variation in Behavior

Activity space size (*r*_adj_ = 0.22 [0.18–0.27]) and activity (*r*_adj_ = 0.34 [0.29–0.41]) were found to be repeatable. Activity space size and activity were correlated at the among‐individual level (*R*_ind_ = 0.36 [0.19–0.52]), suggesting that they form a behavioral syndrome (Table 1).

**TABLE 1:** Summary table of the results of the bivariate model investigating the effects of external, internal, and behavioral drivers on two fine‐scaled behaviors in *Raja undulata* .

Among and within individual (co‐) variances of behaviors							
Behavioral traits		Among individual			Within individual		
		Post. mean	Lower CI	Upper CI	Post. mean	Lower CI	Upper CI
Activity space size	Activity space size	0.202*	0.15	0.26	0.706*	0.679	0.733
Activity	Activity space size	0.087*	0.042	0.139	0.218*	0.199	0.235
Activity	Activity	0.285*	0.216	0.365	0.533*	0.512	0.554

Location effect (effect of explanatory variables on response variable)				
Traits	Post. mean	Lower CI	Upper CI	*p*
Activity space size	0.126	−0.058	0.308	0.174
Activity	0.401	0.195	0.625	< 0.001*
Activity ~ day‐time (day)	−0.927	−0.966	−0.885	< 0.001*
Activity ~ week (1)^+^	4.458	2.429	6.532	< 0.001*
Activity ~ week (2)^+^	11.411	9.161	13.791	< 0.001*
Activity ~ week (3)^+^	−0.567	−2.675	1.55	0.596
Activity space size ~ day‐time (day)	−0.522	−0.568	−0.476	< 0.001*
Activity space size ~ week (1)^+^	−2.975	−5.513	−0.562	0.019*
Activity space size ~ week (2)^+^	−11.952	−14.709	−9.279	< 0.001*
Activity space size ~ week (3)^+^	2.439	0.216	4.932	0.043*
Activity space size ~ disc length	0.107	0.031	0.177	0.005*
Activity ~ disc length	0.103	0.026	0.186	0.012*
Activity space size ~ sex (male)	−0.078	−0.233	0.088	0.349
Activity ~ sex (male)	−0.025	−0.213	0.153	0.8
Activity space size ~ philopatry pattern (SR)	0.064	−0.119	0.248	0.5
Activity ~ philopatry pattern (SR)	−0.013	−0.23	0.198	0.919
Activity space size ~ philopatry pattern (SF)	0.116	−0.118	0.342	0.325
Activity ~ philopatry pattern (SF)	0.122	−0.155	0.401	0.379

*Note:* Iterations = 50,001:999,801, thinning interval = 200, sample size = 4750, DIC: 23,502.52.

Abbreviations: CR, continuous residency; SF, site fidelity; SR, seasonal residency.

^+^ Week is modeled as 3rd order polynomial.

* Statistical significance.

**FIGURE 4:**
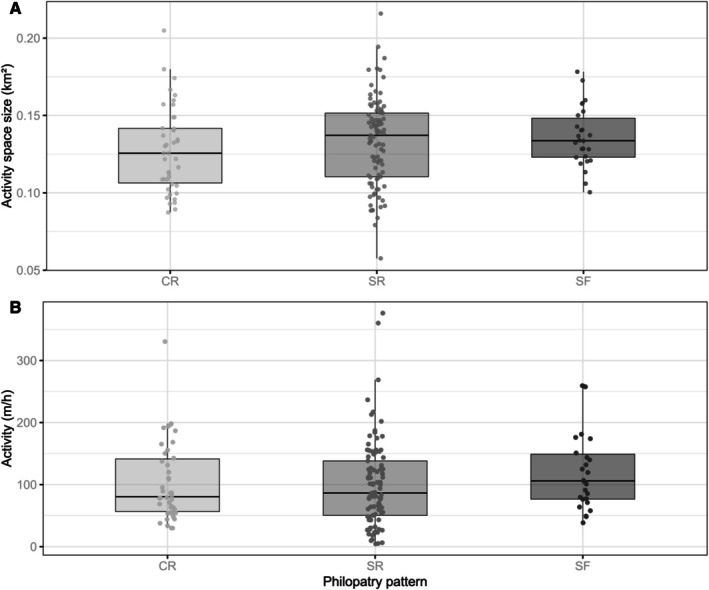
Relationship between the philopatry patterns and (A) activity space size and (B) activity in *Raja undulata* . Black lines represent the group median with 95% error bars, dots represent outliers. CR, continuous residency; SF, site fidelity; SR, seasonal residency.

**FIGURE 5:**
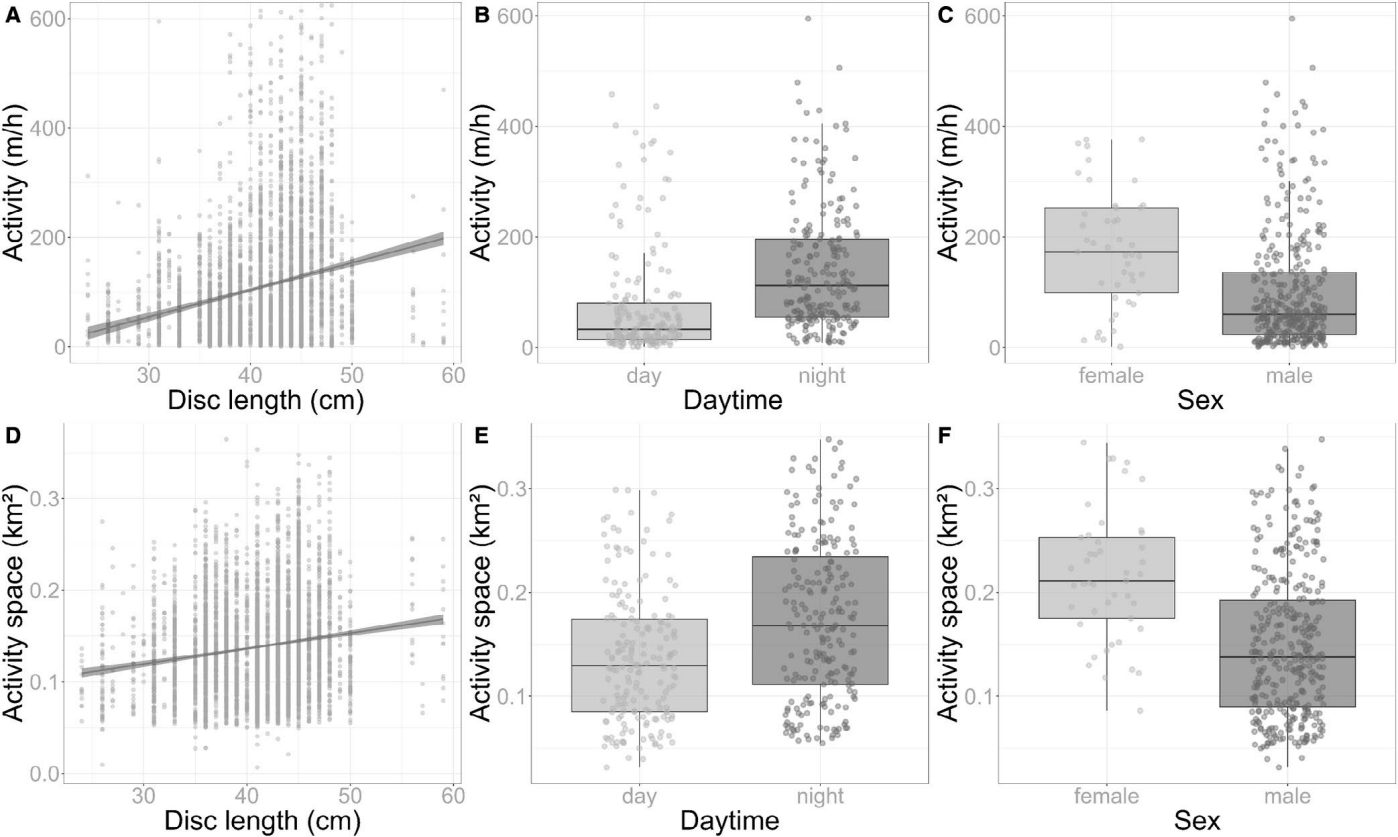
Effect of disc length on activity space size (A) and activity (D) based on smoothers (method = glm) fitted to raw data (gray dots). Effect of daytime (B/E) and sex (C/F) on both behavioral traits. Horizontal black lines represent the group median with 95% error bars, dots represent raw data points (outliers were removed).

We did not find evidence for a significant effect of philopatry on activity and activity space size (Figure 4; Table 1; Annex 2 Figure S6). There was support for a significant effect of temporal and biological variables on activity space size and activity. Week had a statistically significant and strong non‐linear effect on both response variables, acting differently on activity and activity space size (Table 1). Activity increased seasonally with a non‐linear pattern, increasing between November and January (weeks ~47‐03), while the size of activity spaces showed a more complex, s‐shaped seasonal pattern with a notable increase between March and May (weeks ~9–20), thus roughly mirroring previous findings by Leeb et al. (2021). We detected a positive effect of disc length on both behavioral traits, with larger animals being associated with larger activity spaces and higher activity levels (Figure 5A/D). However, the median effect size of disc length on activity was small (0.103 m/h increase per one cm of disc length), suggesting that the effects may not be ecologically significant. The estimated increase in activity between the smallest (24 cm) and largest (59 cm) tagged individuals was only 3.5 m/h. Furthermore, day‐time had a negative effect compared to night‐time, resulting in decreased activity and smaller activity spaces during day‐time (Figure 5B/E). There was no support for a significant effect of sex on activity or activity space (Figure 5C/F).

## 4 Discussion

By combining information about philopatry patterns with high‐resolution movement traits measured on a weekly basis, we investigated whether behaviors occurring at different spatio‐temporal scales are linked with one another in *R. undulata* . We found support for the presence of among‐individual variation in activity and activity space size, but we did not find support for a correlation between those two traits and philopatry pattern. This study represents one of only a handful of studies aiming to understand behavioral syndromes in elasmobranchs (Dhellemmes et al. 2020; Papastamatiou et al. 2022). We further expand on the available information on the philopatry and fine‐scale movement of *R. undulata* and provide pivotal information for understanding the ecology of this commercially exploited species in European waters.

We report the co‐existence of the three philopatry patterns: continuous residency, seasonal residency and site fidelity in *R. undulata* at our study site. To our knowledge, this is the first record of the coexistence of multiple philopatry patterns within a small area for this species, which was previously observed to be seasonally resident in the French Atlantic (Morfin et al. 2019). Among individual variation in philopatry was previously observed in the freshwater whipray (*Himantura dalyensis*) moving in a small lake in southern Sweden (Campbell et al. 2012) and thornback skate ( *Raja clavata* ) in the Thames Estuary and surrounding areas (Hunter et al. 2005). The coexistence of distinct philopatry patterns in this fish population may be maintained through eco‐evolutionary trade‐offs that result in equivalent fitness across behavioral types (Laskowski et al. 2022). From an evolutionary perspective, individual variation in philopatry and migratory behavior may be maintained by two key mechanisms: fluctuating selection, where the optimal strategy shifts over time or space, or life‐history trade‐offs that equalize fitness across strategies (as proposed by the Pace‐of‐life syndrome (POLS) hypothesis) (Laskowski et al. 2022). According to the fluctuating selection hypothesis, different strategies are periodically favored by changing environmental pressures, consequently maintaining variable strategies within a population (Dingemanse and Réale 2013; Laskowski et al. 2022). Alternatively, under the POLS framework, behavioral variation reflects co‐evolved traits, such as growth rate, lifespan and boldness, that balance reproduction and survival and provide equal fitness (Laskowski et al. 2022; Réale et al. 2007). Larger individuals for example, have a more pronounced tendency to migrate, gaining access to areas with greater feeding opportunities and lower predation risk, although this comes with elevated metabolic demands (Mehner and Kasprzak 2011). In contrast, smaller or less healthy fish often remain resident to reduce exposure to predation and conserve energy (Mehner and Kasprzak 2011). Additionally, in many fish species, females have a larger tendency to migrate than males. This pattern likely arises because females gain reproductive advantages with increasing body size (Jonsson and Jonsson 1993), a trait less selected on than small body sizes during migrations (Futamura et al. 2022), whereas males achieve higher fitness by adopting resident strategies (Jonsson and Jonsson 1993). Moreover, variation in large‐scale movement patterns may be shaped by the social niche specialization hypothesis, which suggests that consistent behavioral variation at the individual level arises or increases as a strategy to reduce competition with conspecifics (Montiglio et al. 2013). Consequently, social dominance structures, frequently linked to body size, determine which individuals are compelled or able to migrate, thereby supporting the persistence of both migratory and philopatric behaviors within the population (Chapman, Brönmark, et al. 2011; Reed et al. 2019). Moreover, an exploration–exploitation trade‐off may favor more exploratory, or in our case migratory individuals that access variable or patchy resources (Campos‐Candela et al. 2019; Spiegel et al. 2017), while resident individuals benefit from familiarity with local conditions and reduced (predation) risks. Variable philopatry strategies may also align with personality traits: bold or fast individuals tend to be more likely to migrate (Chapman, Hulthén, et al. 2011; Réale 2010), potentially responding more strongly to spatial or temporal variation in resource availability, whereas shy or slow individuals may thrive in predictable environments, exploiting known resources more effectively (Spiegel et al. 2015). Furthermore, in a population of sea trout ( *Salmo trutta* ) with two repeatable, alternating migration strategies (summer‐ and autumn migrating), for example, migration timing was related to a trade‐off between fitness and risk, resulting in “two evolutionary stable strategies” (Birnie‐Gauvin et al. 2021). Moreover, in salmonids, the coexistence of resident and migratory strategies enhances population resilience by enabling flexible responses to environmental variability, ultimately supporting long‐term persistence and adaptive capacity (Dodson et al. 2013; Horita et al. 2024). Alternative tactics, whether philopatry, migration or dispersal, can buffer populations against changing conditions and contribute to demographic stability over time (Dodson et al. 2013; Horita et al. 2024; Ulaski et al. 2020).

The study site may serve as a node in a network, with individuals visiting primarily in spring and summer before migrating elsewhere, some returning the next season. This hypothesis is supported by recaptures of tagged individuals: while a majority of individuals were recaptured in a radius of < 3 km of the study site, two individuals traveled over ~7 km from the study site towards the southern coast. The maximum observed distance traveled by an individual was ~52 km towards the north. These observations suggest the occurrence of larger scaled movements or even (seasonal) migrations from and to the study site. Additionally, most individuals appeared to ultimately emigrate from our study site, while some specimens returned for all (max. four) detection years. The site's importance to *R. undulata* remains unclear, while other areas that together with the study site may form a connective network are yet to be identified.

This study, to our knowledge, is the first to assess consistent among‐individual variation in behaviors and behavioral syndromes in Rajidae. Behavioral consistency has been reported in previous tracking studies of aquatic species (Alós et al. 2017; Harrison et al. 2015; Villegas‐Ríos et al. 2017). Our results, too, provide evidence for consistent individual differences, with 22% and 34% of the variation in activity space size and activity, respectively, being attributed to among‐individual variation. In a similar study in the batoid *Mobula alfredi*, only 12.9% of variation in presence‐absence patterns could be attributed to among‐individual variation (Andrzejaczek et al. 2020). We found similar or lower long‐term repeatabilities of activity as previous works, for example in *Gadus morhua* (*r*_Activity_ = 0.23/0.34, [0.27–0.4; 0.18–0.28] (Villegas‐Ríos et al. 2017) and *r*_Activity_ = 0.66, [0.58–0.72] (Monk et al. 2023)).

The observed correlation between activity and activity space size suggests that these two traits form a movement syndrome. Our results thus support the occurrence of fine‐scale movement behaviors in *R. undulata* along a movement continuum, which ranges from inactive individuals occupying small activity space sizes to highly active individuals that move through a larger space (within our relatively small study system).

Philopatry pattern, on the other hand, did not explain the levels of activity or the activity space size of individuals. Our results complement previous findings by Harrison et al. (2015), which demonstrated that the fine‐scale behavior is independent of dispersal from the release site in burbot ( *Lota lota* ). Other authors, however, found opposing results, where fish ( *Gadus morhua* , *Rivulus hartii* ) exhibited dispersal‐dependent movement syndromes that included some fine‐scale behavioral traits (Fraser et al. 2001; Villegas‐Ríos et al. 2017).

Bigger fish occupied larger activity spaces, while size did not have an ecologically relevant effect on activity. Our results thus partially contrast previous observations for the species at the study site, which found no effect of body size on either activity or space use (Leeb et al. 2021). Our dataset has expanded since the study by Leeb et al. (2021), as has the size range of tagged animals (previously: 26–48 cm, current: 24–59 cm). Furthermore, *R. undulata* exhibited nocturnal patterns of activity and activity space size at our study site, in agreement with the nocturnal feeding habits (Cabral 2014; Leeb et al. 2021) and resting patterns (Vaudo and Jeremy 2012) reported for this species. The observed rise in activity during autumn and winter was also observed in a previous study by Leeb et al. (2021) as well as in the demersal small‐spotted shark *Scyliorhinus canicula* (Papadopoulo et al. 2023) at the study site. Several mechanisms may underlie this pattern, including internal biological cycles (e.g., reproduction, nutritional need) and environmental factors (e.g., prey availability). *R. undulata* was found to breed during winter and spring in Portugal (Coelho and Erzini 2006; Moura et al. 2007), which may affect activity levels of skates. The site may also be an important foraging ground for the species during this season, as was also hypothesized for *S. canicula* here (Papadopoulo et al. 2023). This idea is further supported by previous studies which found that the diet of *R. undulata* varied drastically among seasons (Moura et al. 2008). Lastly, increased activity may be driven by the species or its preys' nocturnal behavior and the pronounced seasonal difference in daylight hours in Galicia, where longer winter nights extend the active period (Carroll and Harvey‐Carroll 2023; Leeb et al. 2021; Wheeler et al. 2022). We observed no effect of sex on either fine‐scale behavior trait. While sex‐dependent behavior is common in elasmobranchs in for example aggregation, migration, and dispersal (Bird et al. 2020; Portnoy et al. 2015), our results are in accordance with previous observations in *R. undulata* of the same study population that reported no effect of sex on activity or space use (Leeb et al. 2021).

Conservation efforts have recognized the importance of the preservation of behavioral variation between populations and species (Allgeier et al. 2020). However, recent studies highlighted the idea that among‐individual variation of behavior can be an important, yet overlooked component for conservation (Caro and Sherman 2012; Villegas‐Ríos et al. 2021). Individual variation in philopatry patterns and/or movement behaviors has been found in many elasmobranch species, for example *Carcharhinus leucas* (Espinoza et al. 2016), *Carcharias taurus* (Maggs et al. 2019) apart from *R. undulata* and should be accounted for in their conservation (Chapman et al. 2012; Siskey et al. 2019). Potential strategies for protecting different behavioral types include the implementation of dynamic spatial or temporal protective measures, for example seasonal closures or mobile marine protected areas (MPAs), that align with the timing and spatial extent of (partial) migration routes and periods of spatial segregation. The importance thereof was illustrated in the teleost *Gadus morhua* , as individuals exhibiting different movement behaviors encountered different degrees of protection and thus variable probabilities of being harvested as they spent different amounts of “time at risk” outside the MPA borders (Villegas‐Ríos et al. 2021). Furthermore, the expression of alternate life‐history traits such as migration strategies in *Gadus morhua* in the Gulf of Maine, was found to be interlinked with variations in morphological traits that are selected on by fisheries (Sherwood and Grabowski 2010). Consequently, conservation strategies should consider individual variation in philopatry and area use, to protect behavioral diversity.

Furthermore, both consistent among‐individual variation in behaviors and behavioral syndromes might have eco‐evolutionary implications which should be accounted for in conservation and management. Repeatable behaviors are considered to be heritable to some degree (Dochtermann et al. 2015). In an eco‐evolutionary context, heritable individual variation in small‐scaled behaviors provides a basis for evolution to act upon as a response to natural and anthropogenic selection (Uusi‐Heikkilä et al. 2008; Wolf and Weissing 2012). Selective agents such as fisheries, however, may favor individuals that encounter fewer fishing gears due to their movement behavior, for example, those with smaller home ranges (Villegas‐Ríos et al. 2021). Ultimately, this could cause fisheries‐ or protection‐induced selection (Alós et al. 2012, 2017; Thorbjørnsen et al. 2021; Villegas‐Ríos et al. 2017). Similarly, when two or more behaviors are correlated across different conditions and this correlation is genetically determined, these movement syndromes could influence evolutionary processes through the potential co‐evolution of correlated behavioral traits (Dingemanse and Dochtermann 2013; Wolf and Weissing 2012). Selective pressure on one trait would indirectly also select on the other trait(s) in a movement syndrome. On the other hand, if a changing regime requires several traits to shift, and these happen to form a syndrome, this could accelerate evolution (Wolf and Weissing 2012). The presence of a movement syndrome, as that between activity and space‐use in our study, could thus affect species selection, though the observed effect size of this correlation is small compared to similar studies (e.g., *R*_ind_ = 0.98 between movement and home range in *L. lota* , Harrison et al. 2015). The heritability of repeatable behavior and behavioral syndromes in fish is currently poorly understood and movement syndromes could also occur as two traits are expressed in a certain way within the sampled environment, while the same traits might not be correlated in another environment (Dingemanse and Wolf 2013). The lack of correlation between philopatry, measured on a multi‐year basis, and activity and activity space size, both measured on a weekly basis, implies that these traits may evolve independently and suggest that they may be regulated by different mechanisms (Biro and Stamps 2008; Chapman et al. 2015; Dall et al. 2005; Hueter et al. 2004; Tupper and Boutilier 1995). However, although we did not detect a strong link between small‐ and large‐scale behaviors in our study, previous literature provides compelling support for such links (Axling et al. 2023; Malorey et al. 2024; Chapman, Hulthén, et al. 2011; Mettke‐Hofmann et al. 2005), suggesting that both small‐ and large‐scale behavioral traits are relevant for understanding broader movement patterns and their eco‐evolutionary implications. Consequently, the integration of both fine‐ and coarse‐scale data collection (e.g., tracking) and analysis, as well as subsequent use in conservation and management remains essential.

Our study design has several limitations. First, the categorical classification of philopatry patterns may not capture the full complexity of this behavior. An intrinsic bias is present in our classification scheme, as individuals classified as seasonal residents could in truth be individuals expressing either site fidelity or continuous residence, that were tagged only at the end of their stay at the study site before permanent emigration, underestimating the number of resident individuals. Furthermore, our philopatry classification relies on an artificially selected threshold that was based on data and ecological knowledge. However, sensitivity tests suggest any bias is minimal and model outcomes are robust to various assumptions of thresholds. Lastly, the fate and trajectory of animals beyond our telemetry array is unknown, introducing potential bias, as we assume individuals are alive and mobile. This is supported by recaptures of alive animals up to 52 km from the study site, as well as by recent findings combining stable isotope analysis and acoustic telemetry, which showed that in *R. undulata* low‐residence individuals, primarily females, had broader isotopic niche widths, suggesting the use of more diverse habitats and potential roles in connecting distant areas through coastal dispersal (Daban et al. 2024).

By using *R. undulata* as a model species, we have illustrated that multiple philopatry patterns coexist within a population detected in a small area and that behavioral traits exhibited at different spatial and temporal scales occur independent of one another. Selective pressures on philopatry are not expected to drive the co‐evolution of activity levels or activity space. However, our results also demonstrate that due to individual variation occurring at different scales and varying usage of the study site, animals may benefit from conservation measures to different degrees. We believe that the exploration of the relationships between fine‐scale behaviors that shape the daily lives of individuals with movement behaviors that span years and occur at a larger spatial scale is a novel and crucial frontier in movement ecology. While we did not observe this relationship, we emphasize the need for further research in other study systems to identify the conditions under which such connections might emerge and should be incorporated into conservation efforts.

## Author Contributions

**Gonzalo Mucientes:** data curation (supporting), investigation (supporting), methodology (supporting), writing – review and editing (supporting). **Alexandre Alonso‐Fernández:** conceptualization (equal), data curation (lead), formal analysis (supporting), funding acquisition (lead), investigation (supporting), methodology (equal), project administration (lead), resources (equal), software (supporting), supervision (lead), validation (supporting), visualization (supporting), writing – review and editing (supporting). **Kenn Papadopoulo:** data curation (supporting), software (supporting), writing – review and editing (supporting). **David Villegas‐Ríos:** conceptualization (equal), data curation (equal), formal analysis (supporting), investigation (supporting), methodology (equal), resources (supporting), software (supporting), supervision (supporting), writing – original draft (supporting), writing – review and editing (supporting). **Christopher T. Monk:** formal analysis (equal), investigation (supporting), methodology (supporting), software (supporting), writing – review and editing (supporting). **Alina Hillinger:** conceptualization (equal), data curation (supporting), formal analysis (lead), investigation (equal), methodology (equal), software (equal), validation (lead), visualization (lead), writing – original draft (lead), writing – review and editing (lead).

## Disclosure

Statement on inclusion: Our study includes authors from multiple countries, with the majority based in the study site's country. All authors contributed early to the research and study design, to ensure that the diverse sets of perspectives they represent were considered. Relevant literature by local scientists was cited, and collaboration with local authorities and stakeholders was prioritized.

## Conflicts of Interest

The authors declare no conflicts of interest.

## Supporting information


**Data S1:** ece372135‐sup‐0001‐Annex1.xlsx.

**Data S2:** ece372135‐sup‐0002‐Annex2.pdf.

## Data Availability

Data are available from the Flanders Marine Institute (VLIZ) Platform for Marine Research repository: http://www.vliz.be/en/imis?module=dataset&dasid=6523 and European Tracking Network repository: https://marineinfo.org/id/dataset/6605.
