# Supplementary material for: Individual Variation in Philopatry Is Unrelated to Activity and Space Use in the Undulate Skate Raja undulata
Source: Ecol Evol. 2025 Dec 10;15(12):e72135. doi: 10.1002/ece3.72135 (PMC12695698; doi:10.1002/ece3.72135)
Supplement: Supplementary file 2 — Data S2: ece372135‐sup‐0002‐Annex2.pdf. [file ECE3-15-e72135-s002.pdf]

Supplemental Information for:

Individual variation in philopatry is unrelated to activity and space use in the undulate skate *Raja undulata*

Table of Contents:

**Figure S1.** Number of detected skates in the research area per tagging cohort (year) and tagging dates (red dashed lines). The colour of lines indicate the year of tagging.	Page 2
**Table S1.** Results of the sensitivity analysis on the philopatry classification according to imposed absence threshold.	Page 3
**Figure S2.** Results of bi-variate models fit with three absence thresholds, to assess model sensibility to the method of philopatry pattern classification.	Pages 4-6
**Figure S3.** Decision tree, used for the process of interpolating acoustic telemetry data for trajectory estimation in *Raja undulata.*	Page 7
**Figure S4.** Model diagnostics plots (subset).	Pages 8-10
**Figure S5.** Map of recaptures of tagged (acoustically or with t-bar only) individuals of *R. undulata*.	Page 11
**Figure S6.** Posterior estimates of covariation coefficients among the two behaviours (activity, space-use) and predictors in individual *Raja undulata* as estimated by multivariate generalized linear mixed effect models.	Page 12

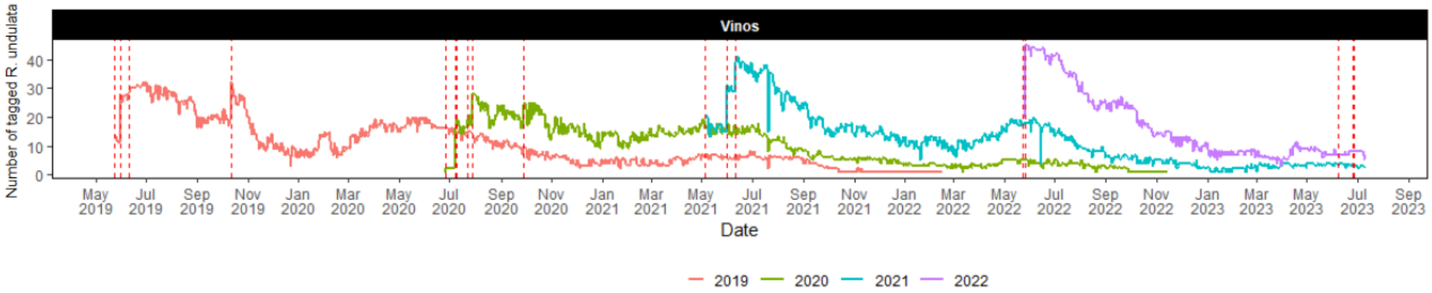

Figure S1. Number of detected skates in the research area per tagging cohort (year) and tagging dates (red dashed lines). The colour of lines indicate the year of tagging.

Table S1. Results of the sensitivity analysis on the philopatry classification according to imposed absence threshold.

	Threshold		
**Philopatry pattern**	60	90	120
Seasonal residency	100	105	109
Continuous residency	35	43	46
Site fidelity	42	29	22

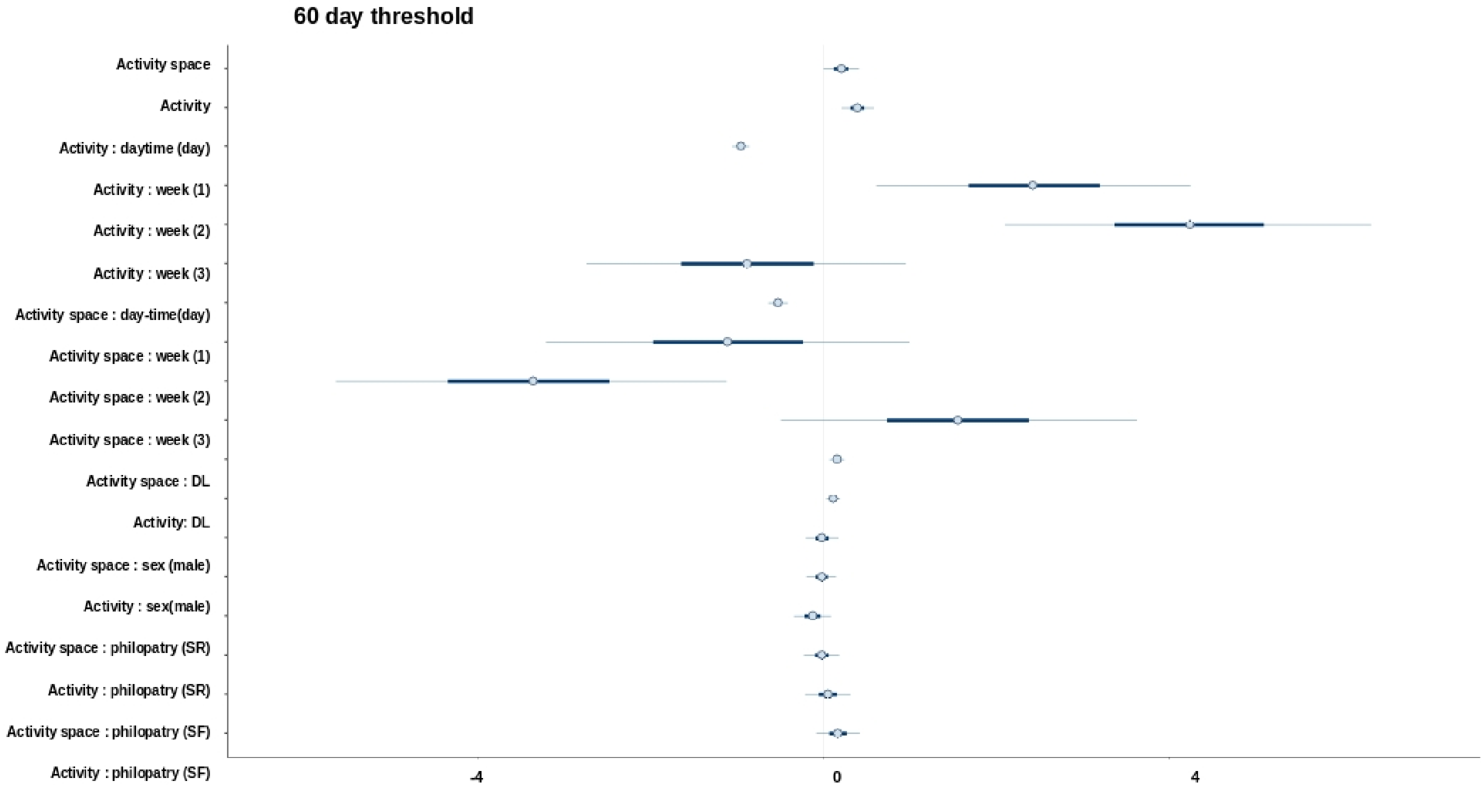

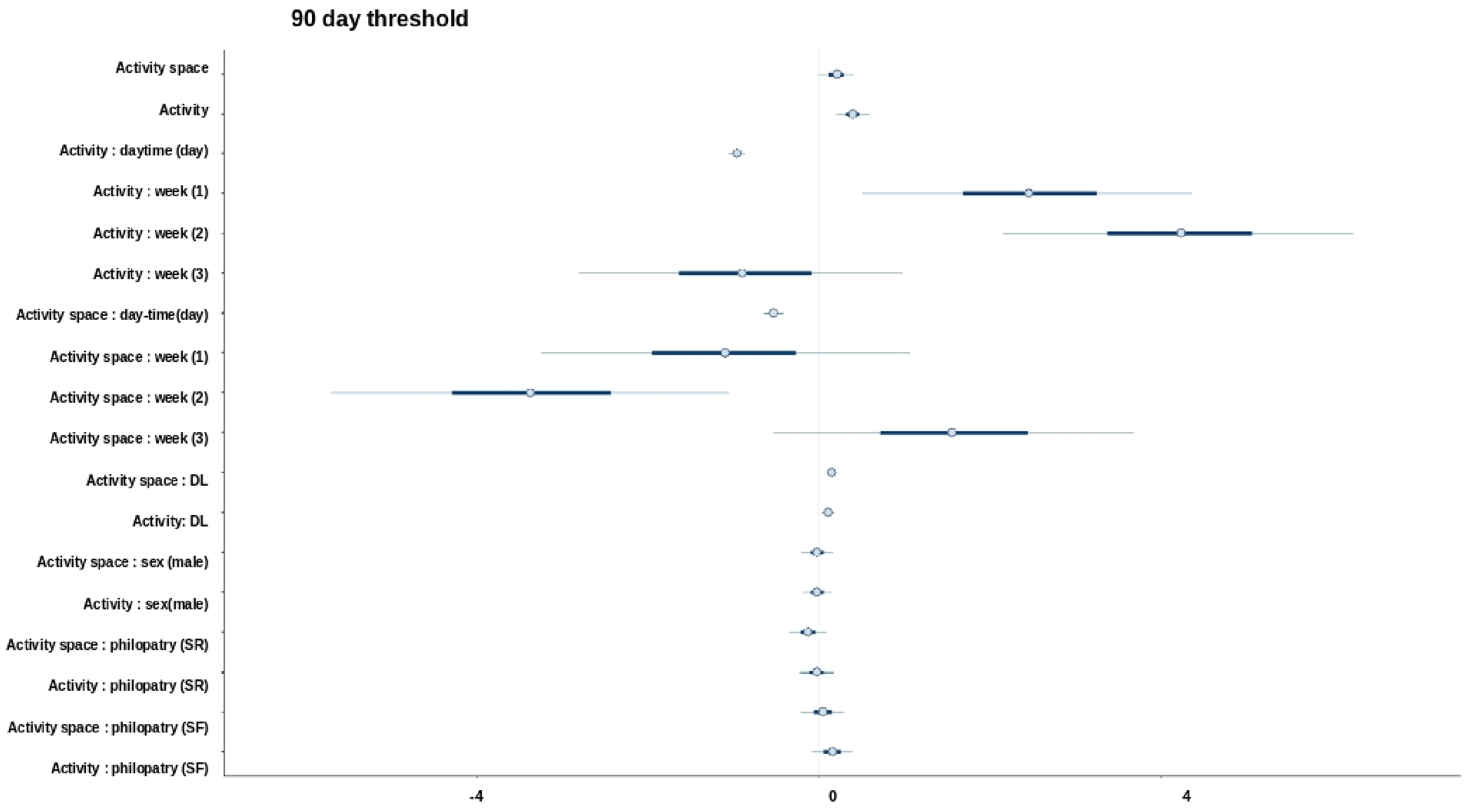
90 day threshold
Activity space
Activity
Activity : daytime (day)
Activity : week (1)
Activity : week (2)
Activity : week (3)
Activity space : day-time(day)
Activity space : week (1)
Activity space : week (2)
Activity space : week (3)
Activity space : DL
Activity: DL
Activity space : sex (male)
Activity : sex(male)
Activity space : philopatry (SR)
Activity : philopatry (SR)
Activity space : philopatry (SF)
Activity : philopatry (SF)
-4
0
4

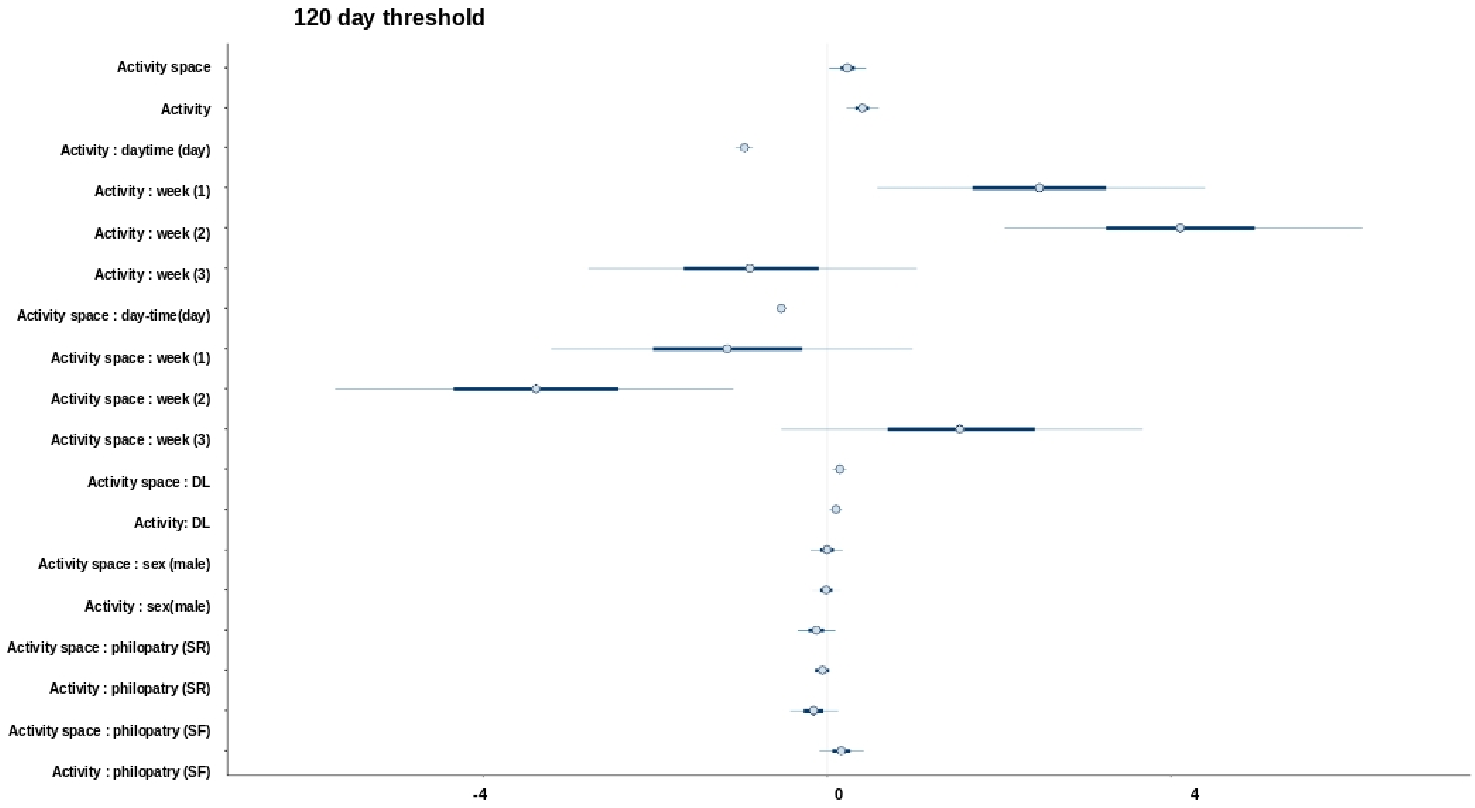

Figure S2. Results of bi-variate models fit with three absence thresholds, to assess model sensibility to the method of philopatry pattern classification. Models were run with 100000 iterations, a burn-in of 50000 and thinning of 200.

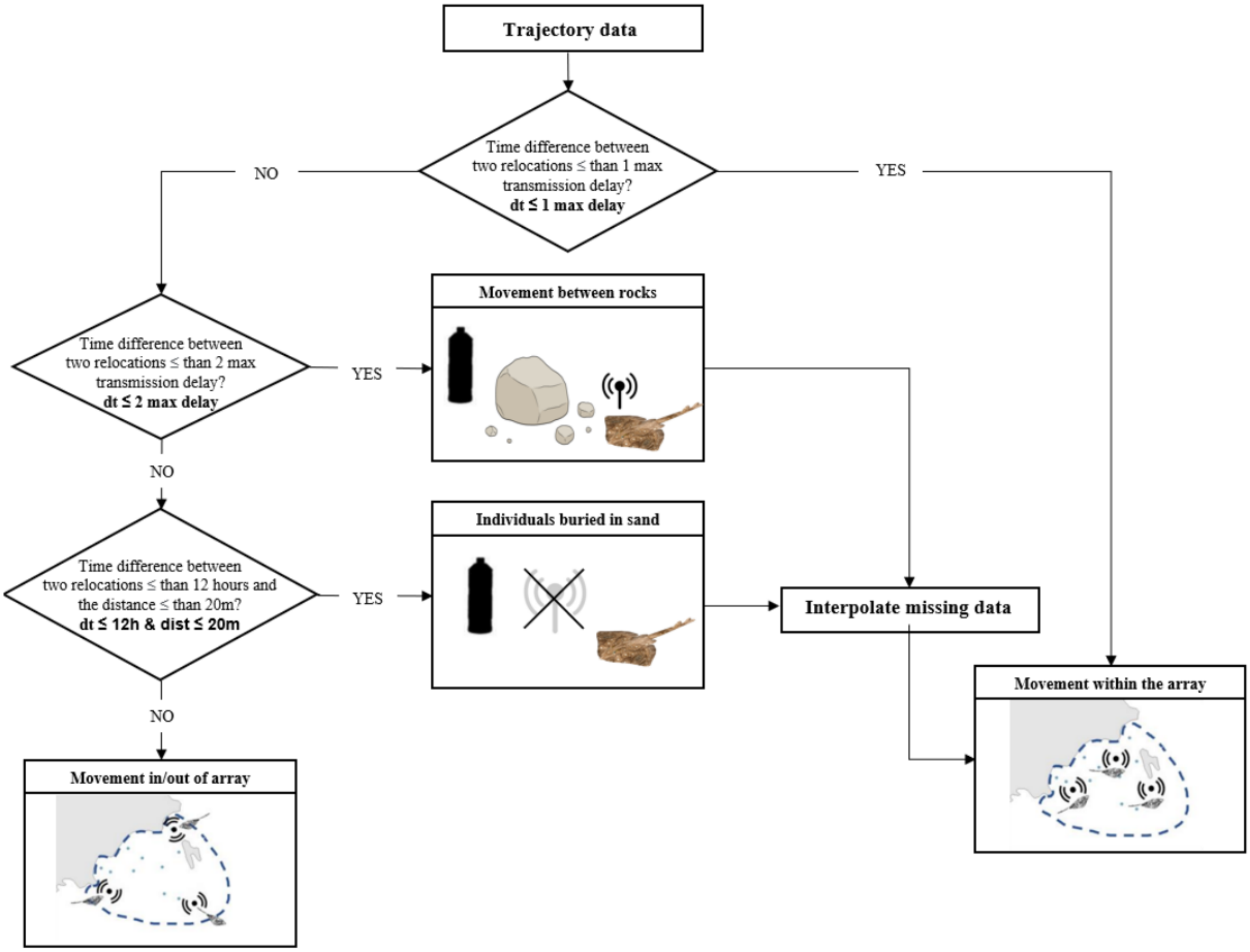

Figure S3. Decision tree, used for the process of interpolating acoustic telemetry data for trajectory estimation in *Raja undulata.* When a trajectory included periods of >12 h without detections, we considered those periods as absences from the study area and data were removed from the analyses; 2) when a trajectory included periods without detections >10 minutes but <12 hours then: 2.1) if the distance between the last position before the time gap and the first one after it was less than 20 m apart, we considered that the fish had remained undetected inside the array (e.g. buried in the sand). Those periods were retained for the analyses as they reflect inactivity periods. 2.2.) if the distance between the last position before the time gap and the first one after was larger than 20 m, we considered that individuals had left the array during the time gap and those data were removed from the analyses. 3) All gaps smaller than 10 minutes were retained as part of the trajectories.

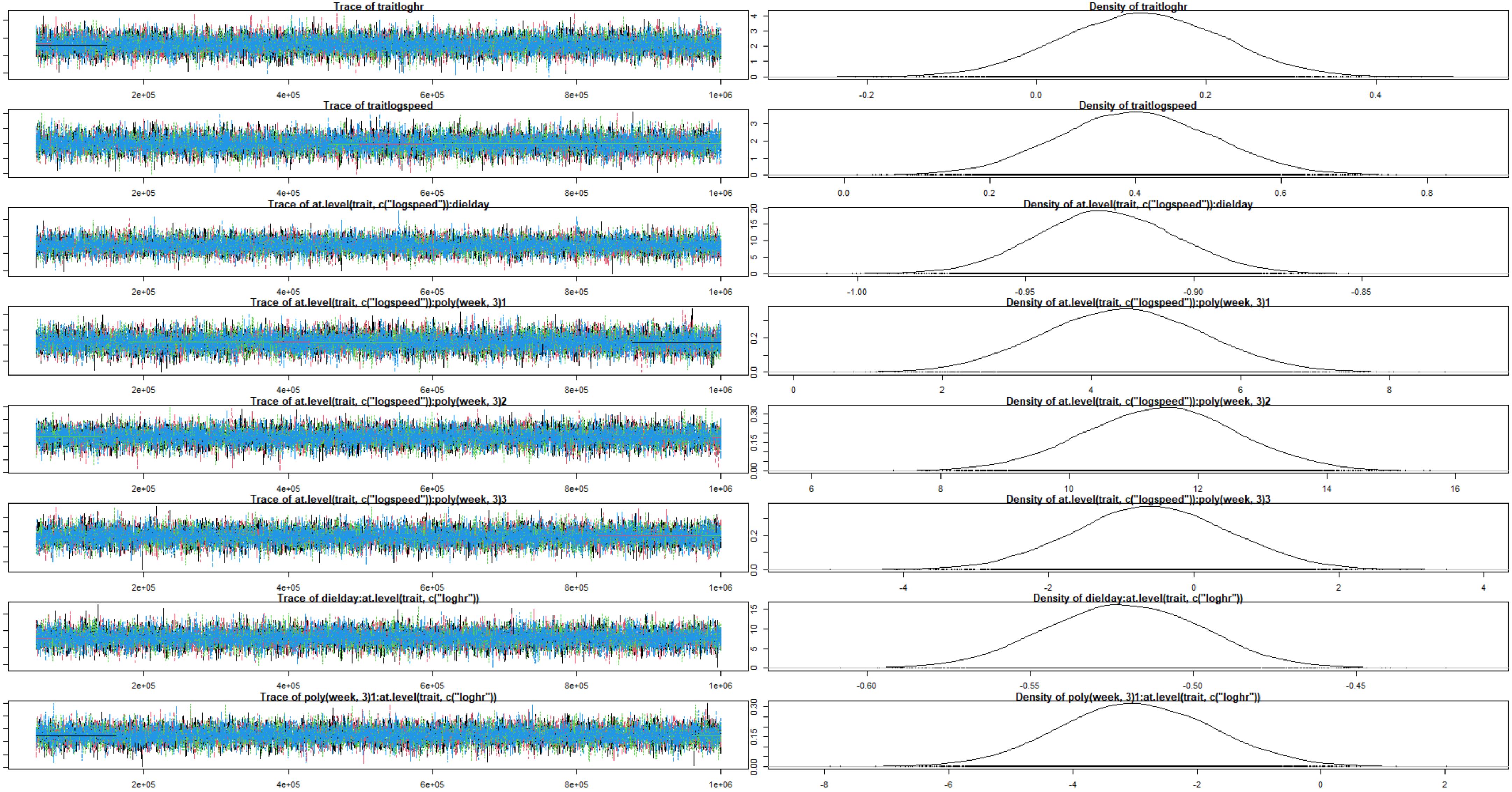
Trace of traitloghr
Density of traitloghr
Trace of traitlogspeed
Density of traitlogspeed
Trace of at.level(trait, c("logspeed")):dielday
Density of at.level(trait, c("logspeed")):dielday
Trace of at.level(trait, c("logspeed")):poly(week, 3)1
Density of at.level(trait, c("logspeed")):poly(week, 3)1
Trace of at.level(trait, c("logspeed")):poly(week, 3)2
Density of at.level(trait, c("logspeed")):poly(week, 3)2
Trace of at.level(trait, c("logspeed")):poly(week, 3)3
Density of at.level(trait, c("logspeed")):poly(week, 3)3
Trace of dielday:at.level(trait, c("loghr"))
Density of dielday:at.level(trait, c("loghr"))
Trace of poly(week, 3)1:at.level(trait, c("loghr"))
Density of poly(week, 3)1:at.level(trait, c("loghr"))

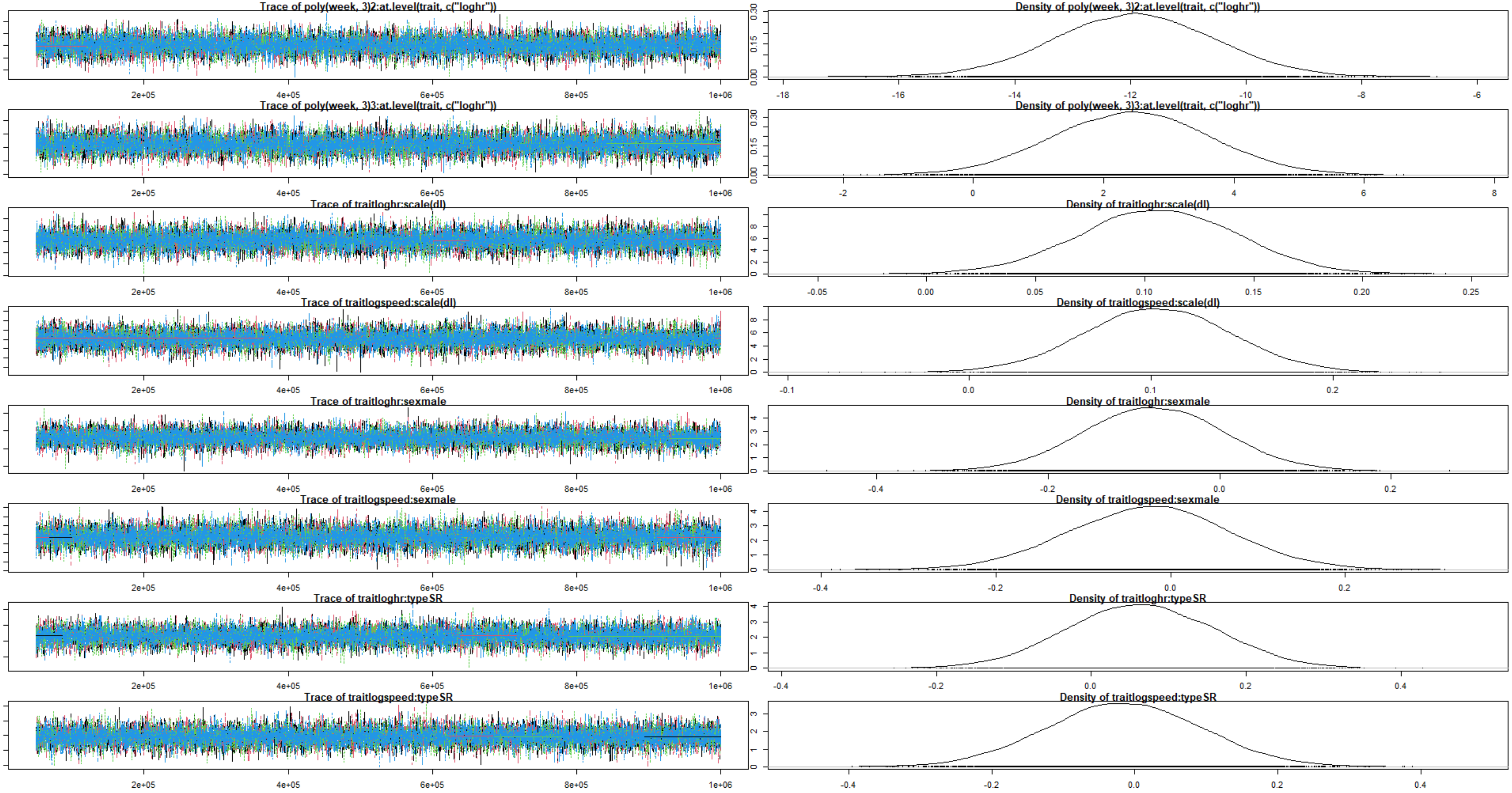
Trace of poly(week, 3)2:at.level(trait, c("loghr"))
Density of poly(week, 3)2:at.level(trait, c("loghr"))
Trace of poly(week, 3)3:at.level(trait, c("loghr"))
Density of poly(week, 3)3:at.level(trait, c("loghr"))
Trace of traitloghr:scale(dl)
Density of traitloghr:scale(dl)
Trace of traitlogspeed:scale(dl)
Density of traitlogspeed:scale(dl)
Trace of traitloghr:sexmale
Density of traitloghr:sexmale
Trace of traitlogspeed:sexmale
Density of traitlogspeed:sexmale
Trace of traitloghr:typeSR
Density of traitloghr:typeSR
Trace of traitlogspeed:typeSR
Density of traitlogspeed:typeSR

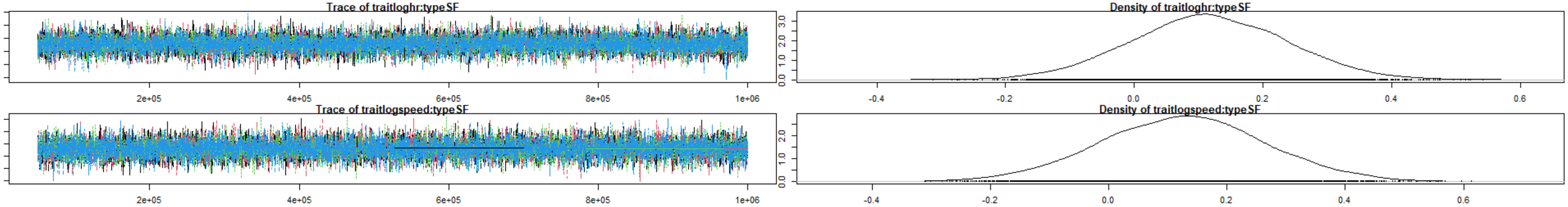

Figure S4. Model diagnostics plots (subset). Left: overview of the state of mixing of four Markov chains (each colour denoting a different chain); right: density plots.

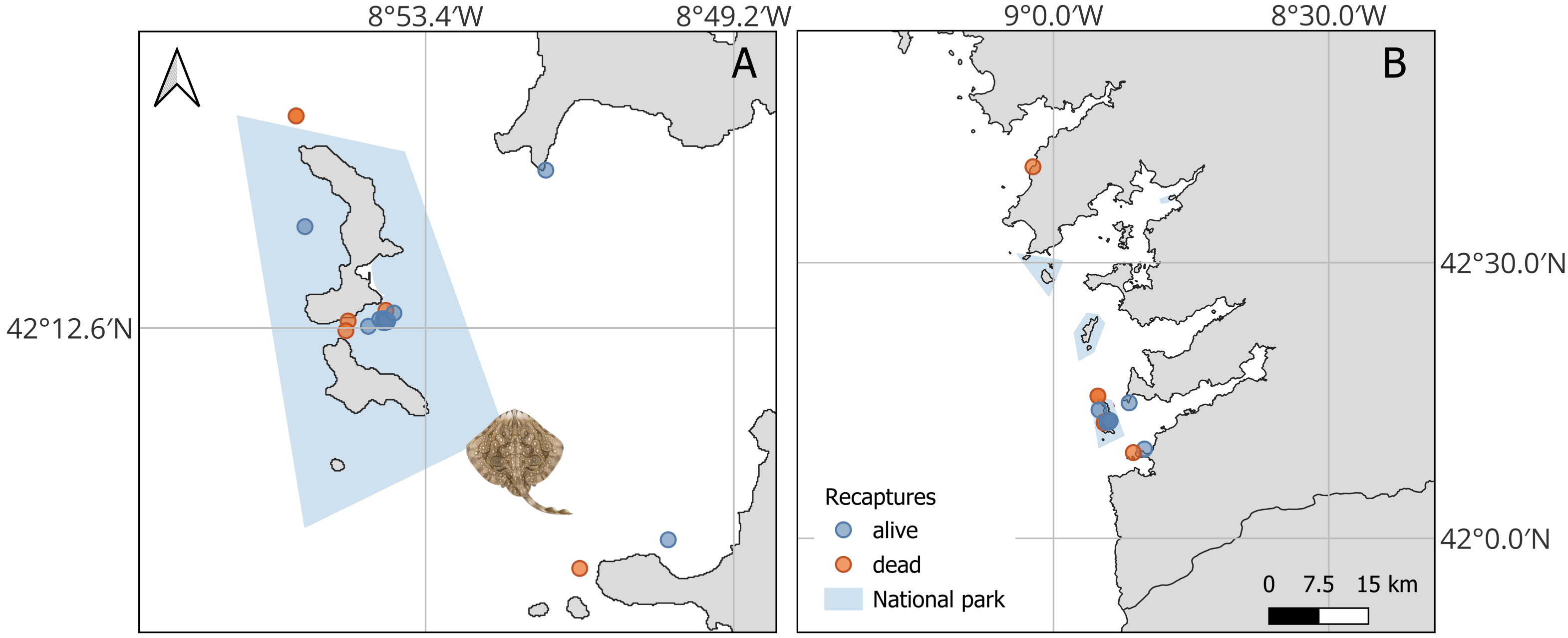

Figure S5. Map of recaptures of tagged (acoustically or with t-bar only) individuals of *R. undulata*. Orange points represent recaptures in which the individual was either encountered dead or killed, blue dots represent recaptures in which the individual was sighted and/or released alive. Blue shaded areas mark the boundaries of regional national parks.

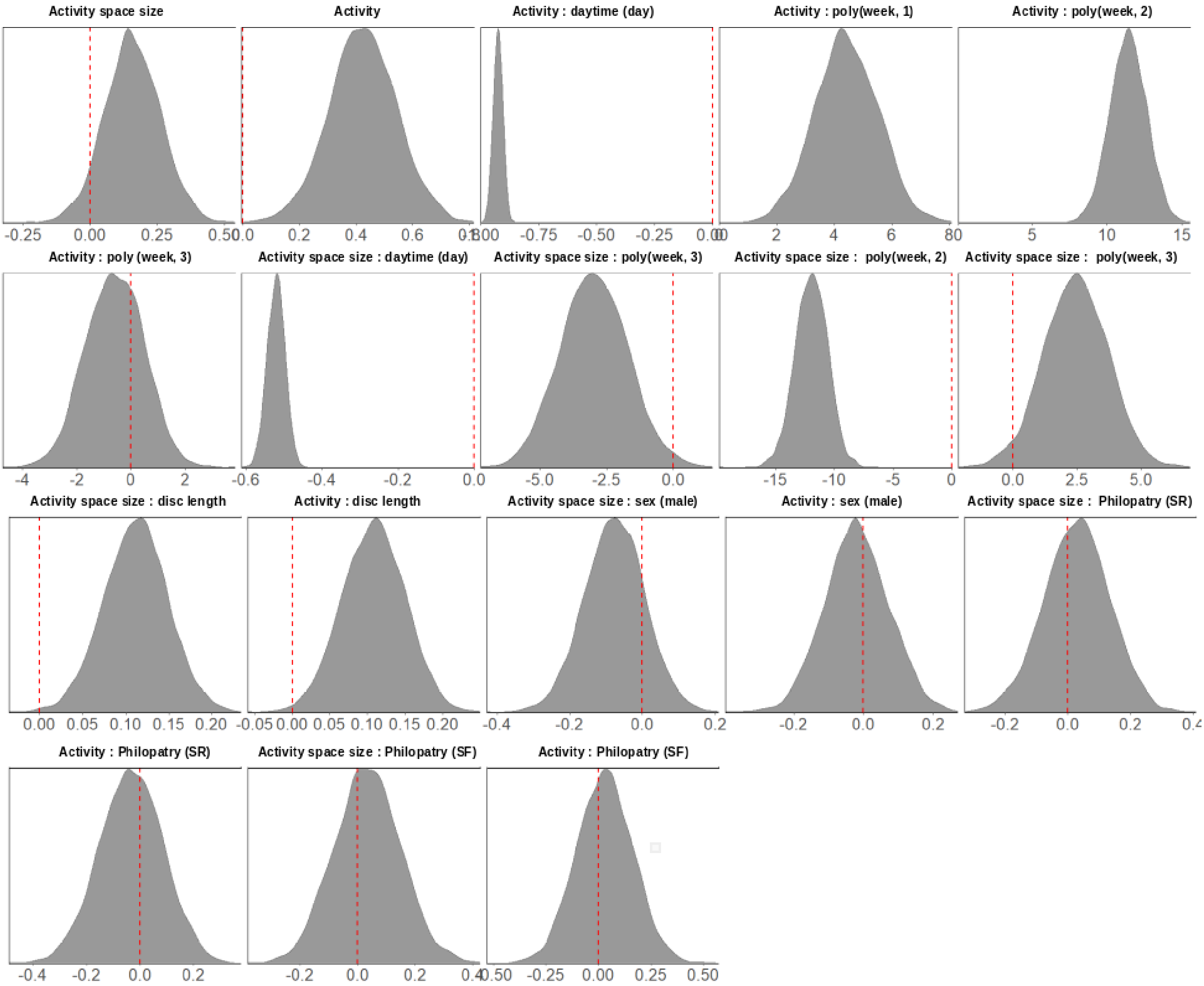

Figure S6. Posterior estimates of covariation coefficients among the two behaviours (activity, space-use) and predictors in individual *Raja undulata* as estimated by multivariate generalized linear mixed effect models. Values are reported on the scale of the corresponding predictor variables.

References

AMIRIX Systems. (2013). *VEMCO Positioning System (VPS) A low cost, non-real-time underwater acoustic fine-scale positioning system*. VPS Datasheet (https://www.oceans-research.com/wp-content/uploads/2016/09/vps.pdf

Leeb, K., Villegas-Ríos, D., Mucientes, G., Garci, M., Gilcoto, M., & Alonso-Fernández, A. (2021). Drivers of spatial behaviour of the endangered undulate skate, *Raja undulata*. *Aquatic Conservation: Marine and Freshwater Ecosystems*, *31*(12), 3466–3479. https://doi.org/10.1002/aqc.3714

Mucientes, G., Leeb, K., Straßer, F.-E., Villegas-Ríos, D., & Alonso-Fernández, A. (2021). Short-term survival, space use and diel patterns of coastal fish species revealed from 'solo datasets.' *Marine and Freshwater Behaviour and Physiology*, *54*(2), 87–95. https://doi.org/10.1080/10236244.2021.1912604

Payne, N. L., Gillanders, B. M., Webber, D. M., & Semmens, J. M. (2010). Interpreting diel activity patterns from acoustic telemetry: The need for controls. *Marine Ecology Progress Series*, *419*, 295–301.
